# Skating into the Unknown: Scoping the Physical, Technical, and Tactical Demands of Competitive Skateboarding

**DOI:** 10.1007/s40279-024-02032-1

**Published:** 2024-05-14

**Authors:** Shelley N. Diewald, Jono Neville, John B. Cronin, David Read, Matt R. Cross

**Affiliations:** 1 Sports Performance Research Institute New Zealand (SPRINZ) at AUT Millennium, Auckland University of Technology, 17 Antares Place, Rosedale, Auckland, 0630 New Zealand; 2 Kitman Labs, Dublin, Ireland; 3 Athlete Training and Health, Houston, TX USA; 4 Skateboarding New Zealand, Auckland, New Zealand

## Abstract

**Background:**

The inclusion of skateboarding in the Olympics suggests that athletes and coaches are seeking ways to enhance their chances of succeeding on the world stage. Understanding what constitutes performance, and what physical, neuromuscular, and biomechanical capacities underlie it, is likely critical to success.

**Objective:**

The aim was to overview the current literature and identify knowledge gaps related to competitive skateboarding performance and associated physical, technical, and tactical demands of Olympic skateboarding disciplines.

**Methods:**

A systematic scoping review was performed considering the Preferred Reporting Items for Systematic Reviews and Meta-Analysis (Extension for Scoping Reviews) guidelines. Data sources were MEDLINE (Ovid), Scopus, SPORTDiscus, and PubMed. We included all peer-reviewed literature after 1970 describing the physiological, neuromuscular, biomechanical, and/or tactical aspects of skateboarding.

**Results:**

Nineteen original articles explored the physiological (*n* = 9), biomechanical (*n* = 8), and technical (*n* = 10) demands of skateboarding. No research explored the tactical demands of competition. Moreover, although competitive males (*n* = 2 studies) and females (*n* = 1 study) were recruited as participants, no research directly related skateboarding demands to performance success in competitive environments.

**Conclusions:**

Ultimately, what constitutes and distinguishes competitive skateboarding is unexplored. There is some evidence indicating aspects of the sport require flexibility and elevated and fast force output of the lower limbs, which may be valuable when attempting to maximise ollie height. Nonetheless, a lack of ecological validity, such as using static ollie tests as opposed to rolling, restricted our ability to provide practical recommendations, and inconsistency of terminology complicated delineating discipline-specific outcomes. Future researchers should first look to objectively identify what skaters do in competition before assessing what qualities enable their performance.

**Supplementary Information:**

The online version contains supplementary material available at 10.1007/s40279-024-02032-1.

## Key Points

**Table:** 

There are no established, objective criteria defining key performance indicators for competitive street or park skateboarding.
While some research exists relating ollie jump height to lower-body power capability, none has related the demands of skateboarding to performance in a competitive environment, in either male or female populations.
Future researchers should first look to objectively identify what skaters do in competition before assessing what qualities enable their performance to ultimately inform more targeted and effective training and development programs for athletes.

## Introduction

In an attempt to mimic wave riding on concrete in the 1950s [1], California surfers created skateboarding, which soon spread as a popular grassroots sport [2]. In 1995, skateboarding gained global mainstream attention with the initiation of the X-Games, leading to its debut at the Olympic Games in Tokyo 2020 [2]. It was subsequently approved for Paris 2024 and Los Angeles 2028 [3]. Skateboarding is one of the fastest-growing sports in the world, with over 50 million people skating globally [4]. These numbers will likely increase with an influx of young athletes seeking to follow the Olympic pathway [5] and succeed on the world stage [6]. To support coaches and athletes in decision-making for training and competition preparation, it is essential to identify key performance indicators (KPIs) and underlying determinants that align with competition success [7].

Olympic skateboarding has two disciplines, “street” and “park” [2], with unique formats and associated judging criteria [8]. In park, athletes perform two or three 45-s runs in which they link a sequence of coping (rail) and aerial tricks together in a “bowl” course [2]. Runs are terminated at any point the skater comes off of the skateboard (i.e. “bails”) [9]. In street, skaters perform isolated tricks on an obstacle (best-trick) and/or a sequence of tricks linked together around the entire skatepark (run), using the kick-push (locomotion with one foot swinging and contacting the ground to propel forward and the other supporting leg on the board) to regulate horizontal speed [9]. In both street and park, tricks can be attempted in various stances (“regular”, “goofy, “switch”, “fakie”, “nollie”) while travelling and rotating either frontside (FS) or backside (BS), and flipping and rotating the board along various axes [10], interacting with obstacles in the environment to create individualised styles [11, 12].

Competitive skateboarding performance is defined by judges, utilising criteria [9] to subjectively rank athletes on their ability to land tricks. The principles employed by judges to compare and rank performances within a given Olympic-qualifying competition round are (1) trick difficulty and variety, (2) execution, (3) use of course and obstacles, (4) flow and consistency, and (5) repetition [8]. The difficulty and variety of performed tricks include obstacle selection, trick selection, and originality and innovation [8]. Common ways of potentially increasing the trick difficulty (and associated score) include performing tricks in different stances (riding “switch”, where the skateboarder rides in the non-preferred stance), linking different variations of tricks (flip trick into a grind), and increasing the height, length, and speed of movement [13]. Although some of these criteria likely have an objective basis through which performance might be targeted and improved (e.g. increased velocity of trick entry) [14], judging ultimately occurs through a subjective lens [8]. So, the relative importance of these factors to creating a good score (i.e. performing well) is a priori unclear.

Drawing from other similar freestyle, subjectively judged, skill-based sports possessing a more substantive body of research (e.g. surfing and snowboarding), we can assume that skateboarding performance depends on an interaction of objective physical, technical, and tactical factors [15, 16]. Within these sports, understanding of these factors is important in providing a basis of empirical data from which to direct training [17], examine athlete progression [16, 18], and enhance athlete performance [19]. Nonetheless, no review on the topic exists, and such information would be best placed in tandem with a thorough understanding of what makes an athlete perform well in situ. A literature review is a critical first step in assessing the current state of the research to determine the most effective path forward to provide practitioners and athletes with objective, evidence-based support to compete at the highest level. To our knowledge, a synthesis of the demands of skateboarding has yet to be conducted.

So, this research aims to evaluate the physical, technical, and tactical demands of competitive skateboarding. It is important to first establish the KPIs of competitive skateboarding, focusing on neuromuscular, physiological, and biomechanical factors essential for high-level performance. A scoping review was selected for this purpose, with the goal of providing a holistic overview of the literature that synthesizes the current evidence on skateboarding performance and qualities, highlights knowledge gaps, and provides guidance for practitioners and future researchers.

## Methods

### Protocol

This study identified and mapped the current literature on the physiological, biomechanical, technical, and tactical demands of competitive skateboarding. The conduct of the scoping review was informed by Arksey and O’Malley’s six stage methodological framework [20], with the protocol conducted according to the Joanna Briggs Institute (JBI) methodology for scoping reviews [21] and reported using the Preferred Reporting Items for Systematic Reviews and Meta-Analysis (PRISMA) Extension for Scoping Reviews: Checklist and Explanation [22]. The final protocol was registered with the Open Science Framework (registration number: 10.17605/OSF.IO/Z94WT).

### Eligibility Criteria

The population, concept, and context (PCC) of interest were defined to form the inclusion criteria [21]. Table 1 presents the final inclusion and exclusion criteria.

**Table 1:** Criteria for inclusion and exclusion in the scoping review

Inclusion criteria	Exclusion criteria
Participants are skateboarders Any competitive, trick-based disciplines (“street”, “park”, “bowl”, “surf”, “vert”, “free-style”) Address physical, technical, and/or tactical demands of skateboarding All ages, sex, and levels of skill Acute and longitudinal study designs English text available Published peer-reviewed literature (including conference proceedings and theses) All study designs After 1970	Participants are “longboarders” (or “downhill”), electric skateboarders, hoverboarders, or any other skating sport (figure skating, roller skating, speed skating) Skateboarding for commuting purposes Social aspects of skateboarding Environmental aspects of skateboarding Mathematical/mechanical modelling of skateboarding (including robotics) Injury-focused studies Non-peer-reviewed (magazines, government documents, conference abstracts) No full text available (after attempted communication with the author)

#### Population

Any populations participating in skateboarding were included, except “longboarders”, “electric skateboarders”, “hoverboarders”, and “disabled populations”. No age, sex, or skill level restrictions were imposed. Trick-based competitive disciplines, “park”, “bowl”, “street”, “vert”, and “freestyle”, were included due to the potential relevance to Olympic disciplines (park and street). We excluded non-trick-based skateboarding (“longboarding”, “downhill”) [12].

#### Concept

Excluding the technological (e.g. equipment) and social demands of skateboarding, research regarding the physical, technical, and tactical demands was included to focus on objective determinants of competitive performance. Cross-sectional and longitudinal studies were included.

#### Context

Only studies that utilised actual participants (skateboarders) were included; explicitly mechanistic studies (e.g. mathematical modelling of the skateboard/rider system) were excluded.

### Information Sources

Databases were the primary information sources. The search was conducted in MEDLINE (Ovid), Scopus, SPORTDiscus, and PubMed on 20 January 2022 by the primary author (SD). An updated search was conducted on 3 May 2023, and three additional articles were identified, with one included in the review. Google Scholar was also searched in incognito mode following the database search for relevant articles [23], and the first 200 titles and abstracts were reviewed for relevance and inclusion. All published information sources after 1970, including full-text theses and conference proceedings, were included.

### Search Strategy

The search strategy was guided by the preferred PRISMA recommendations [24] and aimed to locate published peer-reviewed literature. The primary author (SD) conducted an initial limited search in Google Scholar to identify articles on the topic. Keywords were identified for potential inclusion and exclusion criteria. Inclusion and exclusion criteria were then developed by the primary (SD) and last (MC) authors during the preliminary search and refined before conducting the final scoping review search. The search strategy and Boolean phrases were adapted for each included database and secondary source (Table 2).

**Table 2:** Search databases and associated search strings

Database	Search string
Scopus	Skateboard* [Title, Abstract, and Keywords]
SPORTDiscus	Skateboard* [Title, Abstract, and Keywords]
MEDLINE (Ovid)	Skateboard* [Title, Abstract, and Keywords]
PubMed	"skateboard*"[All Fields]
Google Scholar (secondary source)	Intitle: skateboard OR intitle: skateboarding OR intitle: skateboarder

### Study Selection

Articles were selected per the PRISMA-ScR statement [22], and a modified PRISMA 2020 flow diagram was created to depict the search process. Search results were exported into EndNote [25]. Following duplicate removal, the EndNote library was imported into Rayyan [26] for further screening. The primary author (SD) screened titles for relevance and eligibility. During this stage, articles were removed if they did not relate to the population of interest (e.g. ice, ice hockey, cross country, skating, speed skating, hockey, inline skating, roller skating, cells/animals/soil, carbon monoxide), focused on skateboarding injuries, or were the wrong publication type (magazines, government documents, etc.). The abstracts of the remaining articles were then screened for relevance independently by the primary (SD) and last (MC) authors. Literature was then removed using the exclusion criteria in Table 1. The reference list of articles meeting full eligibility criteria was also screened and examined for additional relevant data and inclusion in the scoping review, termed “snowballing” [27]. Finally, the remaining abstracts were extracted, full-text articles were reviewed independently by the primary (SD) and last (MC) authors, and the exclusion criteria were further applied. All disagreements were resolved immediately during this process, and any excluded full-text records and associated reasoning were reported.

### Data Extraction

The JBI Methodology Guidance for Scoping Reviews was initially utilised to frame the data charting process [23]. The data extraction chart created was an iterative process conducted by the primary author (SD). Key areas of interest, outcome measures, results, and overall findings were identified.

### Critical Analysis and Reporting

The results and discussion sections include an initial descriptive narrative overview of the studies and their relevant findings. A frequency analysis was conducted to provide a numerical summary of the nature, extent, and distribution of the included studies (Tidyverse package, version 1.3.2, in R Statistical Software [RStudio Team, 2020; RStudio: Integrated Development for R; RStudio, PBC, Boston, MA, http://www.rstudio.com/]). Key variables coded to characterise research on demands of competitive skateboarding were publication year and type, study design, study tools, population, and associated demands. Where possible, quantitative results were compared across studies with similar methodologies and subgroups: demand types (physical, technical, and tactical), participation experience level (recreational vs competitive), and competition level (amateur vs professional).

Specifically, the technical demands of skateboarding reported were separated by utilising the 2021 World Skate judging criteria [8]. The difficulty and variety of performed tricks include obstacle selection, trick selection, and originality and innovation. Execution is defined as how well a trick is performed from start to finish. This criterion is further broken down into the quality of trick execution and style of execution, defined by World Skate as: “A distinctive manner or appearance by which a trick is executed, how a skater looks when they do a trick, or how a trick looks when executed. Every skateboarder’s style is unique, and some elements of style (aesthetics, aggression, fluidity, and power) will be subjective to each judge.” [8] World Skate definitions of style elements are presented below in Table 3. According to PRISMA best practice guidance and reporting items for the development of scoping review protocols [21]*,* unlike traditional systematic reviews, scoping reviews do not typically include a step for the assessment of the methodological quality or risk of bias of sources of evidence. Thus, no risk of bias assessment on individual studies was conducted [20, 28].

**Table 3:** Definitions of objective and subjective elements of style within World Skate Skateboarding Judging Criteria

Subjective^a^		Objective^a^	
Fluidity	A *subjective* element of *style* referring to the ease by which an athlete executes the tricks. Fluidity will be subjective to each judge	Speed	An *objective* element of *style* referring to how fast an athlete is going while executing a trick, run, or jam session
Power	*No definition provided*	Height	An *objective* measure of *style* referring to how far off the ground or obstacle an athlete executes a trick/how tall an obstacle is
Aggression	A *subjective* element of *style* referring to bold, forceful, assertive, energetic skateboarding. Aggression will be subjective to each judge	Distance	An *objective* element of *style* referring to how far an athlete travels while executing a trick, be it a grind, a slide, a manual, an air, an ollie, a flip trick, etc
Aesthetics	A *subjective* element of *style* and how a trick looks when executed. For example, foot placement, how the feet catch the skateboard or arm movements An aesthetically good trick is well executed and pleasing to the eye. Aesthetics will be subjective criteria for each judge in both disciplines	Quality of landing	*No definition provided*

^a^‘Subjective’ and ‘objective’ are as defined by World Skate, and do not reflect the technical definitions of objective and subjective criteria. All judging in skateboarding competitions is done through a subjective lens

## Results

### Frequency Analysis

#### Overview

**Fig. 1:**
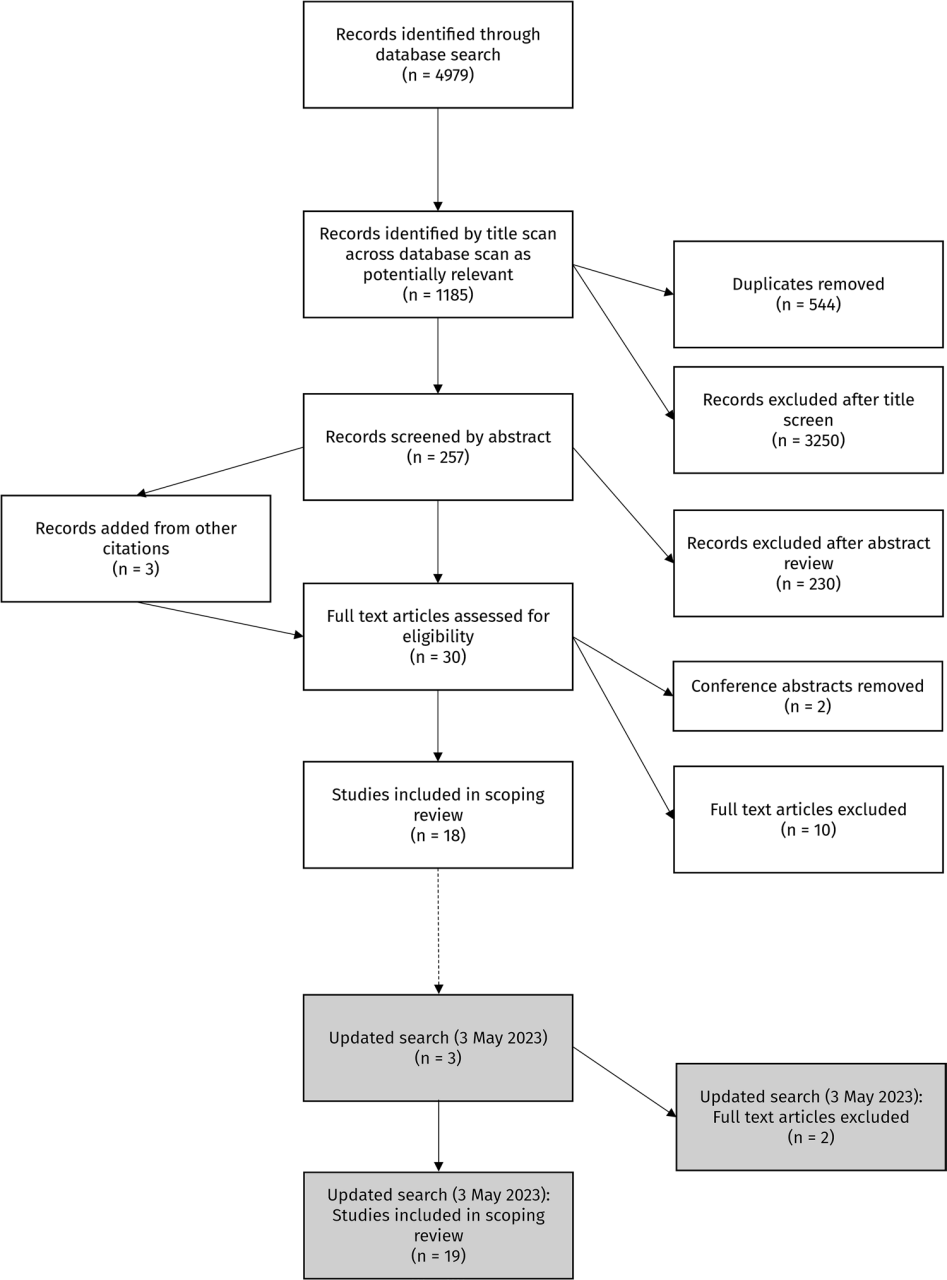
Flowchart of study selection process regarding skateboarding performance

A total of 4979 articles were identified with the search strategy. After 544 duplicates were removed and title screen exclusion criteria were applied, 257 abstracts remained for screening. An additional three studies were identified from the reference list of articles meeting full eligibility criteria. The final title and abstract screening left 30 relevant full-text articles (Fig. 1). Two separate authors reviewed the full-text articles (SD and MC), identifying 18 appropriate studies, utilising the exclusion criteria in Table 1. Following the updated search in May 2023, one additional article was included, resulting in 19 studies for inclusion in the final analysis (Table 4). Excluded full-text records and associated reasoning are included in supplementary Table 2 (a summary table of the excluded studies and associated reasoning for exclusion; see the electronic supplementary material). Articles assessing the physiological (*n* = 9), biomechanical (*n* = 8), and technical (*n* = 10) demands of skateboarding were found; however, tactical demands for competitive skateboarding were not analysed in any included research. Moreover, competitive skateboarders were used as participants (*n* = 3), but the remaining research did not specify participant competition history.

**Table 4:** A table of included studies with participant details, study aim, publication details, relative demands, and study outcomes

Reference^a^	Participant details (number, sex, inclusion criteria, and/or experience)	Study aim	Publication details (type, year) Study design Study location: field-based (Field), laboratory (Lab)-based	Demands^b^			Outcomes (author main findings)
				Biomechanical	Physiological	Technical	
Nessler et al. [39]	*N* = 30 adult skaters “with > 6 months of experience” 10 males (BIPOC only); experience (years): 17.4 ± 11.8 20 females (19 BIPOC); experience (years): 4.3 ± 4.9	Measure and compare heart rate response and activity in female skaters, BIPOC skaters, and non-skaters for 1-h sessions at the skatepark	Journal article (2023) CS, CORR Field		X		Session time (females only): Moving = 57% session Stationary 1–10 s = 5% Stationary 11–60 s = 18% Stationary 1–3 min = 12% Stationary > 3 min = 8% Average heart rate: Females = 138.4 ± 12.0 bpm Horizontal and vertical (elevation) distances travelled significantly greater for males than females and non-skaters
Clark et al. [33]	*N* = 12 male recreational skaters “with > 10 years of experience”	Investigate relationships between asymmetries, subjective skateboarding performance, and objective jump performances in skateboarders	Journal article (2021) CS, CORR Lab		X		Mean SJ asymmetry = 20.5 ± 10.9% Mean CMJ asymmetry = 13.6 ± 9.3% Mean DJ asymmetry = 20.2 ± 16.4% Subjective performance ranking positively correlated with SJ asymmetry, but not CMJ or DJ
Furr et al. [32]	*N* = 71 youth skaters “with > 1 year of experience” 63 males, 8 females; experience (years): 3.0 ± 2.3	Characterise the intensity and duration of youth skateboarders at community skateparks	Journal article (2021) CS, DESC Field		X		Youth recreational skateboarders at community skateparks meet governmental recommendations for exercise and resemble high-intensity interval training
Ou et al. [30]	*N* = 32 amateur and professional skaters “with > 3 years of experience” *N* = 16 professionals (full-time skateboarders with corporate sponsorships) (1 female, 15 male) *N* = 16 amateur (no titles from competitions) (1 female, 15 male)	Investigate ankle joint movement of professional and amateur skateboarders	Journal article (2021) CS, CORR Lab		X		Inversion dominant ankle range of motion of professionals is significantly less than amateurs. No difference in muscle reaction time
Rasid et al. [29]	*N* = 1 male amateur skater “with 5 years of experience”	Identify skateboarding tricks that could be used to identify amateur skaters	Conference proceeding (2021) CS, CORR Lab			X	Ollie and nollie tricks could be suitable for identifying amateur-level skateboarders
Nakashima and Chida [31]	*N* = 1 male recreational skater “with 2 years of experience”	Use simulation to understand ollie mechanics and forces between the rider’s feet and board	Journal article (2021) CS, SIM Lab	X		X	To maximise ollie height, a skater must produce sufficiently fast rotational movement around the rear wheels, by separating both feet from the deck before the tail of the deck hits the ground (pull up front foot early and fast) and separate the rear foot from the deck (to not contact the deck after the pop)
Pietta-Dias et al. [36]	*N* = 11 male professional street skaters (experience level not specified)	Measure knee side-to-side strength asymmetry and compare hamstring:quadricep ratios between limbs of professional street skateboarders	Journal article (2020) CS, DESC Lab		X		No between-limb differences in isometric or isokinetic knee strength. Skaters had weak eccentric hamstring strength relative to quadricep strength
Wiles et al. [35]	*N* = 45 adult skaters “with > 1 year of experience” 44 males, 1 female; experience (years): 3.0 ± 2.3	Investigate if skateboarding at community skateparks would elicit heart rates and durations consistent with government recommendations for cardiovascular fitness in adults	Journal article (2020) CS, DESC Field		X		Adults participating in recreational skateboarding at community skateparks meet government exercise recommendations Average speed = 6.5 ± 1.9 km/h Maximum speed = 19.26 ± 3.44 km/h Average session duration = 65.47 ± 36.17 min Moving = 62% (40 min) Stationary = 38% (25 min) Average distance = 4.58 ± 4.5 km
Wood et al. [34]	*N* = 6 adult male skaters “with > ability to perform tricks”; experience (years): 9.0 ± 4.2	Analyse and compare the kinematics of the ollie in static and dynamic (rolling) conditions	Journal article (2020) CS, CORR Lab	X		X	No significant kinematic differences between static and dynamic ollie (board centre of mass height = 0.24 ± 0.01 m). Higher board height is associated with higher front-foot knee flexion and ankle dorsiflexion
Klostermann and Küng [38]	*N* = 9 adult male “skilled” skaters; experience (years): 14.3 ± 3.6	Quantify eye gaze behaviour of experienced skateboarders and assess relationships with trick difficulty (tricks and obstacles)	Journal article (2017) CS, CORR Field			X	During the approach, the presence of an obstacle caused skaters to shift their gaze from the board to the take-off area in front of the obstacle during both kickflip and ollie. Skaters focused their gaze on board when jumping and landing (kickflip and ollie)
Leuchanka et al. [37]	*N* = 4 “experienced” adult male skaters; experience (years): 3.0 ± 2.3	Quantify take-off and landing forces during static ollie, and up and down a platform	Pilot (2017) CS, DESC Lab	X			Pressure distribution during the take-off and landing centred on the medial forefoot Average peak take-off forces: (ollie down) 2.34 ± 0.32 BWs to (rolling ollie) 2.55 ± 0.51 BWs Average peak landing forces: (static ollie) 2.40 ± 0.33 BWs to (ollie down) 3.15 ± 0.51 BWs
Pham [40]	*N* = 11 adult skaters “with > 1 year of experience” 9 males, 2 females	Investigate how the biomechanics and energetics of skateboarding at various speeds compare to walking and running	Thesis (2016) CS, CORR Lab	X	X		Two distinct push-off styles: brakers and non-brakers Brakers applied more vertical force per stride (average peak vertical force [4 m/s]: brakers = 1.552 ± 0.285; non-brakers = 0.723 ± 0.490) Energy costs when skating at 1.25 m/s like walking (average peak vertical force [1.25 m/s]: brakers = 0.91 ± 0.21 BWs; non-brakers = 0.43 ± 0.32 BWs) Energy costs at 3.0 m/s were half that of running at the same speed (average peak vertical force [3.0 m/s]: brakers = 1.32 ± 0.29 BWs; non-brakers = 0.59 ± 0.43 BWs)
Vorliček et al. [41]	*N* = 10 adult male skaters “with > 4 years of experience”	Compare muscle activity in the ollie and switch ollie	Journal article (2015) CS, CORR Lab			X	Switch ollies required higher back limb muscle activity to maintain knee position. Skaters were unable to control an optimal force in the back leg during the switch ollie (excessive or insufficient force). Skaters had better movement control and a greater range of motion in the front limb during the ollie
Cesari et al. [42]	*N* = 20 adult males (*N* = 8 adult male “expert” skaters “with > 3 years of experience and training > 2 × /week”)	Investigate how participants respond to a sound and simulate an ollie, and whether differences exist between skaters, youth, and adult non-skaters	Journal article (2014) CS, CORR Lab	X		X	Skaters could anticipate and reproduce a jump (shifting bodyweight 200 ms after the sound). Only skaters able to modulate forces under foot and muscle synergies resembling actual jumping
Candotti et al. [43]	*N* = 10 competitive male skaters “with > 2 years of experience” in amateur competitions	Identify relationships between ollie height and lower limb muscle force and power in beginner skaters	Journal article (2012) CS, CORR Lab		X	X	76.5% and 76.1% variance in maximum ollie skateboard explained by CMJ power and body mass, respectively. 50.6% variance explained by the dominant leg, knee extensor isometric strength. Average ollie height = 64.5 cm
Hetzler et al. [44]	*N* = 10 adult skaters “with > 1 year of experience” and ability to skate for 1 h without stopping to rest 8 males, 2 females	Investigate if self-selected skateboarding pace elicits sufficient exercise responses to increase aerobic fitness and maintain a healthy body composition	Research note (2011) CS, CORR Lab and field		X		Estimated energy expenditure at self-selected skating pace for 30 min (using heart rate) = 10.3 ± 3.1 kilocalories/min Total energy expenditure = 308.6 ± 37.9 kilocalories Estimated caloric expenditure for 30 min of continuous skating on a level surface consistent with government recommendations
Determan et al. [45]	*N* = 12 adult “top professional or up-and-coming amateurs” skaters	Quantify ground reaction forces when sliding/grinding down an 8-stair handrail and compare landing and bailing forces	Journal article (2010) CS, CORR Lab	X		X	Impact forces are significantly lower when landing compared to bailing (landing on feet) Maximum vertical forces: Landing = 7.98 BWs Bailing = 12.09 BWs Mean braking forces: Bailing = 2.4 to 5.4 BWs Average horizontal speed at take-off: 4.5 m/s
Nevitt et al. [46]	*N* = 15 adult male “amateur” skaters	Quantify foot forces during the push-off and define frictional requirements of skateboarding shoes	Technical note (2009) CS, CORR Lab	X		X	Three distinct push-off styles: heel-toe (resembling walking), mid-foot, and fore-foot (resembling sprinting). Differences in centre of force between grip tape vs no grip tape Push-off forces: Grip tape = 1054.9 ± 286.6 N No grip tape = 1038.4 ± 273.5 N
Frederick et al. [47]	*N* = 7 adult competitive “top professional or up-and-coming amateurs” skaters	Quantify the kinetics of the ollie and movement patterns during landing	Journal article (2006) CS, CORR Lab	X		X	When skaters ollied off the platform, they intentionally applied a firm landing to stabilise position, creating higher than expected impact force, bore by the forefoot and toes Peak landing forces = 4.52 ± 0.58 BWs at roughly 40–50 ms after contact Pop peak forces = 2.25 ± 0.13 BWs

Data are displayed as mean ± standard deviation, where relevant. *BIPOC* black, indigenous, and other people of colour, *bpm* beats per minute, *BWs* body weights *CMJ* countermovement jump, *CORR* correlational, *CS* cross-sectional, *DESC* descriptive, *DJ* drop jump, *SIM* simulation, *SJ* squat jump

^a^References appear in reverse chronological order

^b^No studies investigated tactical demands, so the column has been removed from the table

#### Publication Details

Articles obtained were published from 2006 to 2023. Most research was published after 2016 (*n* = 11) [29–39], including nine studies published between 2020 and 2023 [39–47]. Journal articles were predominant [30–35, 38, 39, 41–43, 45, 47], with a single conference proceeding (*n* = 1) [29], letter to the editor (*n* = 1) [36], pilot study (*n* = 1) [37], research note (*n* = 1) [44], technical note (*n* = 1) [46], and thesis (*n* = 1) [40].

### Study Design

#### Overview

The search returned only cross-sectional study designs, including descriptive (*n* = 4), correlational study designs (*n* = 14), and a simulation study (*n* = 1) [31]. No longitudinal or training studies were identified.

#### Participant Characteristics

The average number of participants was 17 ± 19 (*n* = 19 studies), ranging from a single-subject design (*n* = 2) [29, 31] to 71 participants (*n* = 1) [32]. The average age of participants ranged from 10.4 ± 2.7 years [32] to 33.3 ± 1.8 years [36]. Participant age was not reported in three studies [37, 46, 47]. Youth participants were included in two studies [32, 39], but only one specifically investigated youth skateboarders (< 18 years) [32]. Where included, the average body mass of adult participants was 65.5 kgs (*n* = 15) and average height ranged from 1.7 to 1.8 m [29–33, 35, 36, 39–41, 43, 44]. Approximately a third of the studies included female participants [30, 32, 35, 39, 40, 44], but only two studies had more than two female participants [32, 39] and only one analysed females separately or reported sex-related differences [39].

“Non-competitive” skateboarders [31–33, 35] and “competitive” skateboarders [30, 43, 47] were used as participants; however, it was not specified in 12 studies whether participants were competitors [29, 34, 36–42, 44–46]. Preferred skateboarding discipline (street or park) was only reported in three studies (i.e. “street”) [29, 36, 43], with preferred competitive discipline only identified in one of the three studies using competing participants [43]. Ten studies adopted experience-based inclusion criteria [29, 30, 32, 34, 35, 39–44].

#### Analysed Movements

All but four studies required participants to skateboard for the research [30, 33, 36, 42]; performing a variety of jumps [29, 34, 37, 38, 41–43, 47], flip tricks [29, 38], grinds [45], and basic locomotion [40, 44, 46]. Only one study investigated a non-flip trick, a grind (or slide) on a handrail [45] (Table 5). When specified, ollies and flip tricks were performed both statically [31, 34, 37] and rolling (while moving) [34, 37, 38, 41, 43, 45, 47]. Vorliček et al. [41] conducted the only study that investigated switch stance manoeuvres. Also, in one study, researchers attempted to use sound to simulate a rolling ollie while participants stood stationary on force plates [42]. Beyond tricks, locomotion-based movement (repeated kick-push), like regulating speed to set up a trick, was also specifically examined for shoe frictional and physiological demands [40, 44].

**Table 5:** World Skate Judging Criteria and relevant studies including study methodology details: trick types, obstacles, objective measures, and any overall outcomes related to skateboarding performance

World Skate Judging Criteria^a^	References	Trick types	Obstacles	Objective measures	Relationship to performance^b^
Difficulty and variety of tricks	[29, 34, 38, 41–43, 45, 47]	Ollie, switch ollie, kickflip, handrail grind, shove-it, nollie, FS 180	Handrail, platform, hurdle	Jump height and muscle activity (EMG)	Greater muscle activity required for switch ollie compared to ollie Altered gaze strategy with increased difficulty of tricks (ollie vs kickflip)
Speed	[32, 35, 39, 44]	Locomotion only (or not specified tricks)	Not specified	Average, minimum, and maximum speeds reached	Not specified
Height and distance	[26, 28, 35, 48–50]	Ollie [31, 34, 37, 38, 43, 47], kickflip [38]	Hurdle, platform	Submaximal (per obstacle height) and maximal board and athlete COM height	Not specified
Quality of landing	46	Ollie	Platform	Foot pressure	Not specified

*EMG* electromyography, *COM* centre of mass, *FS* front-side

^a^The World Skate Judging Criteria most closely related to the article findings and associated objective measures

^b^Skateboarding performance (e.g. greater muscle activity is required to perform more difficult tricks, or a higher jump height is related to a greater competitive score)

#### Skateboarding Equipment

In kinetic skateboarding studies, researchers controlled for skateboarding shoes [37, 45–47] and wheels [40]. In addition, Hetzler et al. [44] required participants to use the same complete skateboard (deck, wheels, and trucks) during locomotion. The remaining studies either did not control any aspect of the skateboard equipment [32, 35, 38, 39] or did not specify [34, 41, 43].

#### Obstacles

Platforms [37, 47], hurdles [38, 41, 43], and handrails [45] were used as obstacles to perform tricks up to [45, 47], off of [37, 45, 47], and/or over [38, 41, 43]. Obstacle heights ranged from a 2-cm hurdle for a switch ollie [25] to a 90-cm-tall handrail [45]. The exact obstacles, heights, and tricks performed can be found in Supplementary Table 1 (see the electronic supplementary material).

#### Laboratory or Field-Based Measurements

Most studies adopted solely laboratory-based measurements of skateboarding [29–31, 33, 34, 36, 37, 40–43, 45–47]. Five studies utilised field-based measurements, in which conditions more closely resembled those typically seen during recreational or competitive skateboarding [32, 35, 38, 39, 44] (e.g. at a local skatepark or over concrete/flat ground). A variety of methods for measuring locomotion in skateboarding were researched: skateboarding on an instrumented treadmill [40], over a force plate [46], and around a concrete track [44].

### Biomechanical Demands

#### Overview

Both kinetics [37, 40, 42, 45–47] and kinematics [31, 34] were quantified and were divided into characterisations of locomotion [40, 46] and tricks [31, 34, 37, 42, 45, 47].

#### Landing

Two journal articles and a pilot study specifically focused on the landing aspect of tricks [37, 45, 47]. Pressure sensing insoles [37, 45, 47] and force plates [45, 47] were used to quantity impact forces when landing from an ollie (36 cm, 45.7 cm) [37, 47] or eight-stair (2.13 m) handrail grind/slide [45].

### Physiological Demands

#### Overview

Nine studies reported on the physiological demands of skateboarding [30, 32, 33, 35, 36, 39, 40, 43, 44]. Both aerobic [32, 35, 40, 44] and anaerobic [30, 33, 36, 43] demands were quantified in laboratory [30, 33, 36, 40, 43] and field [32, 35, 39, 44] conditions. Three studies attempted to detect associations between physiological measures and presumed skateboarding performance metrics such as ollie jump height [43], subjective performance ranking [33], and career status/experience [30]. Only three studies included skateboarding tricks and obstacles when assessing physiological demands [32, 35, 39], and no research was found that specifically quantified the physiological demands of specific or consecutive skateboarding tricks.

#### Aerobic

Five research studies investigated the physiological aerobic demands of skateboarding [32, 35, 39, 40, 44] in adult [35] and youth [32] populations. Aerobic demands of tricks [30, 33, 37] and locomotion were assessed [40, 44].

#### Anaerobic

Skateboarders of various skill levels were tested for physiological strength [36, 43], power [33, 43], and flexibility [30]. Only the lower limbs were researched, specifically isometric hip extension [43], and isometric [43] and isokinetic knee flexion and extension [36]. Lower-limb power was assessed using unilateral [33] and bilateral jump tests [43]. Asymmetries in lower-limb strength and power were also analysed [33, 36]. No female strength and power data were measured. Only one study assessed the stability, balance, and range of motion of professional and amateur skateboarders [30].

### Technical Demands

#### Overview

Ten studies investigated the technical demands of skateboarding skills [29, 31, 34, 38, 41–43, 45–47]. None investigated consecutively performed skateboarding tricks, and as such, no evidence was found on “flow and consistency” or repetition. Furthermore, no studies were found investigating subjective measures of style. Thus, this scoping review only captures World Skate's objective “execution of style”: speed, height, distance, and quality of landing of single trick attempts (Table 5).

No technical studies included female participants, and competitive skateboarders participated in only two technical studies [43, 47]. Also, only one technical study collected data outside the laboratory environment [38]. Kinetics [42, 45–47] and kinematics [31, 34] were quantified, and technologies used to understand the technical demands of tricks included electromyography (EMG) [41, 42], motion capture [31, 34] or video recording [38, 41], force plates [42, 45–47], a load cell [43], an eye-tracking system [38], and an inertial measurement sensor (IMU) [29].

#### Difficulty and Variety of Tricks

The successful execution of tricks was not related to competition. Most literature investigated the ollie manoeuvre [29, 34, 38, 41–43, 47], with one study simulating an ollie without a moving skateboard [42]. Researchers also investigated presumably more difficult tricks, such as the switch ollie [41], kickflip [29, 38], and grind [45].

#### Speed

No evidence of speed being measured (athlete [horizontal, vertical, or rotational speed] or board rotational speed [flip speed]) during specific skateboarding tricks to relate to performance metrics was found. Although Determan et al. [45] reported approach speed (4.5 m/s), no other horizontal or vertical speed of the skateboarder before or after landing was reported. The speed of locomotion was measured in four studies [32, 35, 39, 44]; however, only three measured speed in typical skateboarding environments (e.g. at a local skatepark with obstacles) [32, 35, 39]. No locomotion speed in a competitive setting or with competitive skateboarder participants was reported.

#### Height and Distance

Measures of height included maximum ollie board height determined by the maximum obstacle height cleared [43], obstacle-defined height [37, 38, 47], maximum athlete centre of mass height [34], and maximum board height [31, 34]*.* Trick heights were measured during static and rolling conditions, and measured using motion caption [34], force plates [31], and by obstacle height [37, 38, 41, 43, 45, 47]. Distance-related metrics such as rail length, grind time, or take-off and landing distance were unmeasured.

#### Quality of Landing

A single study [45] measured pressure under the soles and visually assessed landing strategies; however, the quality of landing was not related to either subjective or objective performance.

## Discussion

### Overview and Main Findings

A scoping search of peer-reviewed literature was conducted to (1) identify the physical, technical, and tactical demands of competitive skateboarding, (2) synthesise the findings of the peer-reviewed literature, and (3) highlight limitations and gaps in the literature to guide future research directions. No research explored the tactical demands of competitive skateboarding. Surprisingly, although competitive athletes were used as participants, no research existed relating the demands of skateboarding to performance in a competitive environment. The literature is dominated by laboratory-based measurements of fundamental, isolated skateboarding tricks (e.g. kinetics and kinematics of the ollie). Moreover, the inconsistency and lack of skateboarding terminology further complicated the ability to synthesize findings for practical outcomes. Thus, all research included in the review and subsequent discussion on findings related to performance is presumptive about what constitutes and distinguishes competitive performance in skateboarding.

### Study Design

Although various cross-sectional study designs were utilised to quantify the physical and technical demands of skateboarding, the lack of standardisation or consistency in terminology rendered comparing findings between groups (e.g. sex, age, skill level, discipline) and synthesising across studies difficult. The two Olympic skateboarding disciplines, park and street, vary in format and trick selection. Like other freestyle sports with multiple disciplines (freestyle vs downhill snowboarding), the skills (presumably) required to perform them likely differ [51, 52]. Of the few studies that defined participant skateboarding styles, associations with skateboarding performance were unexplored.

We suggest future research should specify the preferred skateboarding discipline of participants, to ensure sample group findings are applicable and representative of the wider population. Moreover, consistent terminology should be adopted when describing intra-participant characteristics, such as the preferred skateboarding stance (left vs right foot, front vs back foot, dominant vs non-dominant). We recommend that authors clarify both the preferred skateboarding stance (goofy or regular) and dominant leg (leg which athletes would prefer to kick a ball) [33]. This should ensure all tricks and their associated difficulty, such as switch tricks, can be consistently and correctly compared.

Along similar lines, equipment use, standardisation, and subsequent reporting were inconsistent. The degree to which this might influence observed results and interpretation is unknown, but ground reaction forces and joint kinematics in similar sports (e.g. freestyle snowboarding) are known to be sensitive to equipment design and choices (e.g. boot wear, binding angle) [53]. In skateboarding, studies measuring landing impacts would likely be affected by the wheels’ hardness and the trucks’ tightness, resulting in potentially a high amount of uncontrolled variance [45]. Nonetheless, addressing this in research could be complicated since skateboarding equipment and set-up (e.g. truck tightness) are highly individual to the skater’s preference [9]. Requiring all participants to use the same equipment may not be feasible or ecologically valid. So, researchers should attempt to control equipment in other ways, such as intra-participant normalisation [54].

A lack of consensus on the performance calibre of skateboarders was also evident. Most researchers utilised unreliable time-based metrics to define participants’ level of training and skill. Participant skill level was presented both objectively [29, 31–34, 38–43] as “years of experience”, and subjectively [29–33, 35–39, 42–47], referencing the level of experience (“recreational”, “amateur”, “professional”, “skilled”, “highly skilled”, “experienced”, “expert”, and “competitors”). Learning in action sports is very individualistic [6], with likely high movement skill transfer from one freestyle board sport to another [55]. Specifically, Künzell and Lukas [55] found skateboarding lessons to facilitate learning to snowboard, challenging the notion that more “years of experience” equates to a higher skill level. A standardised framework to identify the training and performance calibre of skateboarders is necessary for research to follow the basic principle of specificity [49]. A robust and objective definition of skateboarding cohorts would allow comparison between and within studies. We propose that future skateboarding researchers utilise an approach per McKay et al.’s recommendations [49].

Skateboarding performance is assessed using subjective scores allocated during judging. The scores are intended to differentiate the placing of skateboarders [8], rather than act as a highly sensitive instrument to reflect the specific magnitude of performance difference [49]. Thus, we recommend competitive skateboarding research should rely on proximal rankings from governing bodies (e.g. World Skate) to classify participant skill levels.

#### Ecological Validity of Tests Used

Ollie jump height was used most as the KPI by academics exploring physical and technical demands (Table 5 and Supplementary Table 1). The reason for this selection is understandable, as anecdotally the more height a skater can achieve, the larger the potential obstacles they can utilise, or could allow more airtime to perform flips and rotations of the board. Both presumably would increase trick difficulty and associated score, although importantly, this remains unexplored. Nevertheless, how ollie jump height was measured varied greatly (static vs rolling). Notably, the difference in testing severely limits the ability to compare findings across studies.

Studies that utilised force plates during the ollie either constrained take-off or landing point [31, 34, 38, 45, 47]. While understandable due to laboratory limitations and standardisation practices, imposing these constraints on the skaters may have altered technique and resulted in sub-maximal heights. Along similar lines, neither take-off nor landing speed were reported during ollie jump tests. The speed before take-off likely greatly affects technique and ability to gain maximal height [56]. Thus, by limiting the distance to take-off, these tests potentially measured the optimal technique for that specific scenario only, instead of maximal capability. Although authors reported both maximum board [34, 37, 38, 43, 46, 47] and athlete jump height [34] during the ollie, most jump heights reported were actually the minimum height as determined by the obstacle used [37, 38, 43, 47]. Candotti et al. [43] measured maximal ollie jump height by raising a hurdle height with each successful attempt at clearing the obstacle. Although arguably a more ecologically valid approach, the sensitivity was limited to 5-cm increments [41]. Future research should specify the construct and metrics assessed (maximal vs submaximal rolling or static height) and design the test accordingly. This is a defined criteria in street and part skateboarding used by judges to distinguish performances [8], so researchers should utilise established reliable technology, such as video or in-shoe sensors [34], to measure board height.

### Demands

#### Physiological

The physiological demands of park skateboarding were not specifically addressed in the literature. In street skateboarding, Furr et al. [32] and Wiles et al. [35] found that skating for an hour at the skatepark mimicked heart rates and intensity intervals of gym-based high-intensity intermittent training. While unclear, this seemingly included all flip tricks, rest periods, changes in elevation, and the use (or not) of obstacles. Average adult speeds while moving in the skatepark (6.5 ± 1.9 km/h) [35] were comparable to those controlled by Pham [40] (4.5 and 10.8 km/h) when investigating the energy requirements of the kick-push. Locomotion speeds used by Hetzler et al. [44] (17.05 km/h) were more comparable with top speeds reached by both youth (17.19 ± 3.92 km/h) [32] and adult (19.26 ± 3.44 km/h) [35] skaters.

No studies accounted for skateboarding session variation (tricks attempted, tricks landed/bailed, utilisation and height of obstacles, types of tricks, etc.). Since bailing a trick seems to incur significantly greater impact forces than landing [45], the physiological demands on each skater likely depend on session characteristics. For example, we speculate that an hour of attempting a jump from an eight-stair handrail would almost certainly require greater mechanical demands than an hour session at the skatepark with occasional submaximal ollies on flat ground. We suggest future researchers should aim to quantify the trick details of skateboarding sessions to understand the physiological demands of various skateboarding styles and disciplines (street and park). This could have spanning implications for coaching, specifically for load management strategies related to injury risk [57].

The high-intensity, intermittent nature of recreational street skateboarding [32, 35] shows some similarities to competitive surfing [58]. However, surfers must recover quickly during short rest periods (20-s paddling periods, followed by stationary 10-s periods), which does not appear to be the case in skateboarding, with much longer rest periods between runs and best-trick attempts [9]. Adult recreational skateboarders spent 18% of their session stationary for over 1 min [35]. Although this may not reflect competitive skateboarding sessions or competitions, it would seem from the current evidence that skateboarding is less aerobically demanding than surfing, with skateboarding activity typically lasting less than 45 s, in both park and street competition run formats [8]. Also, rest periods between competitive runs typically range from 3 to 8 min [9]. Thus, skateboarding physiological demands may more closely resemble freestyle snowboarding, where aerobic fitness does not appear to significantly determine performance, and the rest periods between runs are similar [59]. Regardless, where aerobic fitness may be advantageous for training and recovery, anaerobic fitness probably has greater direct performance-related benefits for skateboarders [59].

Skateboarding research that investigated the anaerobic demands focused solely on the lower body. When compared to “performance”, Candotti et al. found that power in the countermovement jump (CMJ) could explain 76.3% of ollie jump height [43]. Also, 50.6% of ollie height could be explained by knee extensor muscle strength of the dominant limb (typically the back foot) [41]. Amateur competitive skateboarders achieved jump heights of 35.3 ± 4 cm and 44.4 ± 6.3 cm in the squat jump (SJ) and CMJ, respectively. Similar CMJ heights were achieved by elite freestyle snowboarders (32.5 to 48.9 cm) and Olympic male volleyball players (44.5 cm) [59, 60]. Interestingly, CMJ height was considered a significant determining factor between selected (49 ± 5 cm) and non-selected (42 ± 7 cm) elite male competitive surfers [61]. Lower body dynamic strength production is likely also important to skateboarding, particularly during the “pop” preceding most street tricks [31]. Unfortunately, no studies in skateboarding compared anaerobic capabilities and associated outcomes, such as CMJ height (or even ollie jump height), to competitive performance success (e.g. scores within a run or competition standings). Thus, the relationship of these factors to better performance is speculative.

Due to the asymmetrical nature of skateboarding, some hypothesised that unilateral anaerobic capacities could be important [33]. However, no significant between-limb differences have been observed [33, 36], nor were between-limb asymmetries in jump power clearly detrimental to the performance (jump height) of experienced, recreational skateboarders [33]. So, although the evidence is weak, there appears to be an importance of bilateral lower body strength and power underlying skateboarding performance [31, 43]. Yet counterintuitively, performance may be less sensitive to strength and power asymmetries [33, 36], potentially due to skateboarders performing tricks in different, more demanding stances (switch, fakie).

Increased ankle dorsiflexion range of motion allows athletes to handle the forces during aerial landings [62], and greater dorsiflexion in the front ankle appears associated with a higher ollie jump height [34]. Similarly, in surfing, greater ankle dorsiflexion range of motion was a distinguishing performance factor [62]. Professional skateboarders reported similar ankle dorsiflexion range of motion values (front foot = 43.50 ± 7.47°; back foot = 42.00 ± 7.75°) to competitive surfers (front foot = 43.0° ± 8.2°; back foot = 42.6° ± 7.2°) [30]. Therefore, while speculative, greater ankle dorsiflexion range of motion, especially in the front foot, could be related to improved performance and reduced injury risk.

#### Biomechanical

Research primarily focused on the kinematics and kinetics of aerial tricks and associated landings. Vertical landing ground reaction forces ranged from 4.52 ± 0.58 [47] to 7.98 body weights (BWs) [45] in the static ollie and handrail grind/slide, respectively. Comparatively, surfers typically experience up to 6 BWs of force during aerial landings [15], and big air slopestyle skiers experience about 2 BWs of force during landings [63]. Determan et al. [45] also measured vertical forces up to 12 BWs when the skater deliberately bailed and landed on their feet rather than on top of their board. Higher impact forces are thought to contribute to injuries in gymnastics, where athletes hit up to 14 BWs of force [64], when, like skateboarders, they deliberately “stick” the landing [64]. Thus, skateboarders may benefit by adopting strategies to cope with the repetitive high forces experienced during trick landings to improve the quality and minimise the risk of injury.

Although the biomechanical skateboarding studies included in this scoping review measured similar trick landing forces, there were conflicting results on force application points from the pressure insole sensors used [37, 45, 47]. Also, there was no clear agreement regarding the location of force application during skateboarding locomotion [40, 46]. So, although forces experienced by skateboarders can be high (relative to other similar sports), both take-off and landing styles and techniques likely impact the force applied and attenuated. While training methods have been implemented in various skill-based board sports to enhance landing technique [65], the impact of such training on skateboarding style (specifically, landing quality) and subsequent competitive performance remains unknown. For instance, as previously mentioned, skaters frequently achieve a clean landing by deliberately exerting additional pressure on the board upon touchdown [45]. Although this elevates the forces involved [45], potentially increasing the injury risk [66], it could enhance trick execution or even positively influence the judges’ perception of style and landing quality [67, 68]. The association between take-off and landing techniques and forces during tricks, and more broadly performance, should be investigated.

#### Technical

Klostermann and Küng [38] found a strong link between specific task demands and visual information processing. Including an obstacle altered the fixed visual attention (gaze) strategy of “skilled” (14.3 ± 3.6 years of experience) male skateboarders. When attempting to ollie over a 20-cm hurdle (12.5-cm hurdle for a kickflip) compared to a rolling ollie with no obstacle, skateboarders shifted their gaze during the approach from looking at the skateboard (34.4% [no obstacle] vs 16.8% [obstacle] of approach time), to focus on the area in front of the obstacle. In addition, when required to perform more technical tricks without an obstacle (e.g. kickflip), skateboarders focused on their skateboard longer than when performing an ollie. After the take-off, neither the trick difficulty (kickflip vs ollie) nor the obstacle appeared to affect gaze behaviour; all skateboarders directed their gaze to the board for landing [38]. Thus, gaze strategy adjustment due to obstacles may indicate a change in trick difficulty, a key judging criterion [8]. The only obstacles utilised in the research were platforms [45], hurdles [38, 41, 43], and a handrail [45], but no studies included tricks performed on ramps, quarter pipes, or inclined surfaces. Hence, future research should include and define more obstacles when assessing skateboarding performance and related underlying demands, despite there being no understanding of the relationship between obstacle choice and competitive success.

In addition to the gazing strategy being an indicator of difficulty, the shifting of body weight and musculature activity may also indicate increased trick difficulty [42] and differentiate performance. Attempting tricks in switch stance required increased muscular strength and coordination of the lower limbs [41], suggesting that skaters must produce force quickly by shifting their body weight and maintaining balance by evenly distributing force across the lower limb muscles. It would seem skaters utilise lower-limb muscle coordination to cope with high landing forces while maintaining stylish elements.

In addition to a capacity to cope with high forces, lower limb flexibility also appears to be an important skill when attempting and landing tricks at height [47]. In addition to flexing their ankles, knees, and hips to dampen the load and find balance on the board [47], skaters flexed their lower limbs in the air to obtain a higher board height, important when trying to clear obstacles [34]. This was echoed by Nakashima and Chida [31], who suggested the importance for the skateboarder to pull up the front foot “early and rapidly” during the take-off stage of the ollie. Moreover, the amount of force (strength) applied to the board during the pop was less important to maximum jump height than the speed at which that force is applied [47], suggesting the relationship between strength and speed influences technique. Therefore, as anticipated, skateboarding appears a technical sport, requiring athletes to produce force effectively and efficiently and have enough lower limb range of motion to obtain maximum height and dampen landing forces.

#### Tactical Demands

The subjectivity of skateboarding renders it complicated to objectively determine what distinguishes and constitutes success. Compared to traditional sports with objective winning differentiators (e.g. athletics), skateboarders are ranked each round; how judges determine these rankings, and whether they are consistent within and across competitions, is unknown. Yet, no published research to date explores the tactical demands of competitive skateboarding, and thus coaches are left to speculate how to support skaters to be successful at competitions. Furthermore, while an attempt has been made to explore and interpret findings on various demands, their practical utility remains unclear without knowing how these relate to competition performance. This sets a clear prioritisation for future research direction to explore the tactical demands of competition and then the underlying capacities required.

### Summary and Recommendations for Future Research

Skateboarding is an individual, skill-based sport, and performance presumably relies on essential physiological, biomechanical, technical, and tactical skills to achieve success [15, 16]. Although previous research, as reported in the scoping review, attempted to understand various skateboarding demands, there is a general lack of standardisation and thorough reporting across studies, restricting synthesis. More importantly, it is imperative to acknowledge that the term “performance” is frequently used despite lacking empirical evidence establishing the criteria for a truly successful skateboarding performance (or competition). Thus, findings from this review and associated literature are speculative. To address this critical gap, future research must first look to objectively identify the tactical demands of competitive skateboarding, before attempting to assess methods of differentiating and improving performance.

Future research must consider the ecological validity of study methodologies when concluding findings. Study design should be standardised and reported, including equipment (e.g. standardisation of shoes), metrics selected (e.g. board height vs athlete height), technology (video analysis), analytical approaches (e.g. determination of jump height calculations), and terminology. Skateboarding, like traditional sports, is inextricably linked to the environment [6], evidenced by the communities’ high values of creativity and free-nature culture [69]. For example, it is rare for athletes to compete solely on flat ground; all Olympic-qualifying street skateboarding competitions are performed in a “skatepark” with various obstacles, inclinations, and surface types [9]. Thus, research should not remove aspects of the environment and competition constraints that are critical to understanding performance. This may be a challenging undertaking, given the trade-off between ecological validity and standardisation (accuracy); a certain degree of control is required to reduce variance to draw conclusive findings from the study [6]. As such, careful consideration should be taken when designing a study to ensure accuracy, without sacrificing the applicability to the real world—a challenge for all applied sport science research. Also, as for many traditional sports, the skateboarding literature suffers from an underrepresentation of females [69]. Future skateboarding research should explore both male and female skaters to effectively improve performance and reduce injury.

## Conclusion

This scoping review identified large gaps in the skateboarding literature, with few studies using competitive skateboarders, and inconsistent terminology complicated the ability to delineate discipline-specific outcomes. There are some data suggesting certain aspects of the sport require quick and high force output of the lower limbs and draws on anaerobic energy sources. Most research focused on quantifying isolated tricks, with lower-limb power potentially valuable when attempting to maximise ollie height, and indications that flexibility might be a factor. Nonetheless, effectively no research investigated tactical demands, which renders the practical utility of the current research questionable, since it is presently unclear what constitutes and underlies an objectively better performance in street and park skateboarding. Thus, skateboarding appears a technical sport requiring athletes to produce force effectively and efficiently, utilising lower-limb muscle coordination to cope with high landing forces while maintaining stylish elements.

### Supplementary Information

Below is the link to the electronic supplementary material.

Supplementary file1 (DOCX 54 KB)

## Declarations

### Data Availability

All data generated or analysed during this study are included in this published article and its supplementary information files.

### Code Availability

All code used to create the results tables are available by request.
