# Supplementary material for: Skating into the Unknown: Scoping the Physical, Technical, and Tactical Demands of Competitive Skateboarding
Source: Sports Med. 2024 May 14;54(6):1399–418. doi: 10.1007/s40279-024-02032-1 (PMC11239769; doi:10.1007/s40279-024-02032-1)
Supplement: Supplementary file 1 — Supplementary file1 (DOCX 54 KB) [file 40279_2024_2032_MOESM1_ESM.docx]

**TITLE PAGE**

**Title of Article:** Skating into the unknown: Scoping the physical, technical, and tactical demands of competitive skateboarding

**Submission Type:** Review Article

**Authors, Institutional/Corporate Affiliations, ORCID and email:**

Shelley N. Diewald^1,2^, ORCID: 0000-0003-0348-017X, shelley.diewald@aut.ac.nz

Jono Neville^1^, ORCID: 0000-0001-8826-6839, jono.neville@aut.ac.nz

John B Cronin^1,3^, ORCID: 0000-0002-8889-0911, john.cronin@aut.ac.nz

David Read^4^ , david@skateboarding.nz

Matt R. Cross^1^, ORCID: 0000-0003-1579-3720, matthew.cross@aut.ac.nz

^1^Sports Performance Research Institute New Zealand (SPRINZ) at AUT Millennium, Auckland University of Technology, Auckland, New Zealand

^2^Kitman Labs, Dublin, Ireland

^3^Athlete Training and Health, Houston, Texas, USA

^4^Skateboarding New Zealand, New Zealand

**Contact Details for the Corresponding Author:**

SHELLEY N. DIEWALD

17 Antares Place

Rosedale, Auckland 0630

New Zealand

Shelley.diewald@aut.ac.nz

+64 9 921 9001 ext.26517

**Preferred Running Head:** Physical, technical, and tactical demands of skateboarding

**Supplemental Material:**

2 Tables

**STATEMENTS AND DECLARATIONS**

Shelley Diewald is supported by an academic scholarship from High Performance New Zealand (HPSNZ). The authors have no professional relationship with a for-profit organisation that would benefit from this study. The research results do not constitute an endorsement by the authors or the journal.

# SUPPLEMENTARY MATERIAL

Table 1: A summary of included studies and relevant skateboarding-specific methodological and study design information

| **Reference** | **Study Design Details (Skateboarding Style, length of sessions, isolated tricks, etc.)** | **Environment**  **(Concrete, Lab Floor, Skatepark, etc.)** | **Equipment**  **(Shoes, Wheels, Deck, Trucks, etc.)^[[1]](#footnote-1)^** | **Tricks**  **(Type, Static or Rolling)** | **Obstacles**  **(Type, Height)** | **Performance Assessments** | **Constraints** |
| --- | --- | --- | --- | --- | --- | --- | --- |
| Nessler, Lundquist [33] | 1-hour skate sessions, presumably street-skateboarding | Local Skatepark | Not controlled for or measured | Included, but not controlled for or measured | Included, but not controlled for or measured | None | None |
| Clark, Bishop [27] | General skateboarding^[[2]](#footnote-2)^ | Not Provided | Not Provided | Not Provided | Not Provided | Subjectively ranked by lead author on ability to land difficult tricks consistently | Not Provided |
| Furr, Nessler [26] | 1-hour skate sessions, presumably street-skateboarding | Local Skatepark | Not controlled for or measured | Included, but not controlled for or measured | Included, but not controlled for or measured | None | None |
| Ou, Chen [24] | No skateboarding involved | Not Applicable | Not Applicable | Not Applicable | Not Applicable | Not Applicable | Not Applicable |
| Rasid, Kamarudin [23] | Isolated, street-skateboarding tricks | Laboratory Floor | Not controlled for or measured | All tricks presumably static  Ollie  Kickflip  Shuv-it  Nollie  FS 180 | None | None | Constrained by the size of the motion capture space (not provided) |
| Nakashima and Chida [25] | Isolated, street-skateboarding tricks | Laboratory Floor | Not controlled for or measured | Static Ollie | None | None | Tricks performed on a force plate (size not provided) |
| Pietta-Dias, Ruas [30] | No skateboarding involved | Not Applicable | Not Applicable | Not Applicable | Not Applicable | Not Applicable | Not Applicable |
| Wiles, Kellogg [29] | 1-hour skate sessions, presumably street-skateboarding | Local Skatepark | Not controlled for or measured | Included, but not controlled for or measured | Included, but not controlled for or measured | None | None |
| Wood, Oliveira [28] | Isolated, street-skateboarding tricks | Laboratory Floor | Not Provided | Static Ollie  Rolling Ollie | None | Board and skater COM height | Participants required to “pop” on a designated mark, 3 meters after the start of a 10-meter-long walkway |
| Klostermann and Küng [32] | Isolated, street-skateboarding tricks | Outdoor Carpark (Flat, refurbished pavement) | Not controlled for or measured | Rolling Ollie  Rolling Kickflip | 20cm hurdle (ollie)  12.5 cm hurdle (kickflip) | None | Started from standing at position marked by a cross, then kick-push 12 meters in a straight line into the jump zone (4 meters long x 3 meters wide) |
| Leuchanka, Ewen [31] | Isolated street-skateboarding tricks | Laboratory Floor | Standardised shoes | Static Ollie  Rolling Ollie  Ollie Down | 36 cm tall platform (length unknown) (ollie) | None | Length of the platform constraining the take-off of the ollie down the platform |
| Pham [34] | Flat-ground locomotion | Treadmill | Standardised wheels | Kick-Push | None | None | Width and speed of the treadmill |
| Vorlíček, Svoboda [35] | Isolated street-skateboarding tricks | Concrete | Not Provided | Rolling Ollie  Rolling Switch Ollie | 20cm hurdle (ollie)  2 cm hurdle (switch ollie) | None | 25 meters of track |
| Cesari, Camponogara [36] | No skateboarding involved | Not Applicable | Not Applicable | Not Applicable | Not Applicable | Not Applicable | Not Applicable |
| Candotti, Loss [37] | Isolated, street-skateboarding tricks | Laboratory Floor | Not Provided | Rolling Ollie | Hurdle in 5cm increments | Max hurdle height cleared | None |
| Hetzler, Hunt [38] | Flat-ground locomotion | Treadmill  Flat-ground concrete track | Standardised completes (deck, truck, wheels) | Kick-Push | None | None | Width and speed of the treadmill |
| Determan, Frederick [39] | Isolated, street-skateboarding tricks | Laboratory Floor | Standardised shoes | Handrail Slide or Grind | 8-stair handrail  (2.13m high)  (62° from horizontal, 0.91 meters above flat at start, 0.90m from flat at bottom) | None | Landing constrained by force plate at the bottom of the hand rail  (1.2 meter long, 0.48 meters from the bottom of the stairs)  Take-off constrained by 8.2 meter flat ground platform leading to handrail |
| Nevitt, Determan [40] | Flat-ground locomotion | Laboratory Floor | Standardised shoes | Kick-Push | None | None | Direction constrained by embedded force plate (size not provided) |
| Frederick, Determan [41] | Isolated, street-skateboarding tricks | Laboratory Floor | Standardised shoes | Ollie Up  Ollie Down | 46.7 cm tall platform (length unknown) | None | Landing and take-off constrained by force plate placed 80 cm beyond the platform end |

Table 2: A summary table of the excluded studies and associated reasoning for exclusion

| **Title** | **Author(s)** | **Publication Year** | **Publication Type** | **Reason for Exclusion** |
| --- | --- | --- | --- | --- |
| Human control of the skateboard | Hubbard, M. | 1980 | Peer-reviewed article | Not relevant for this review. Describes the skater for use in a mathematical model, but no technical, tactical, or physical components |
| Comparison of physiology and biomechanics speed skating, cycling, and skateboard exercise | Kandou TW, Houtman IL, vd Bol E, de Boer RW, de Groot G, van Ingen Schenau GJ. | 1988 | Peer-reviewed article | Not relevant for this review. The skateboarding described is not trick-based skateboarding. There is also no access to the full text article. |
| The physiological responses to skateboarding | Mestek, M. | 2001 | Thesis | No access to the full text article |
| Impact forces during skateboarding landings. (Abstract) | Determan, J. and Frederick, E. C. and Cox, J. | 2004 | Conference Proceeding / Abstract | Quality of evidence lacking. Only a conference proceeding and abstract |
| Kinetics of the skateboarding kickflip | Determan, J. and Frederick, E. and Cox, J. and Nevitt, M. | 2006 | Conference Proceeding / Abstract | Quality of evidence lacking. Only a conference proceeding and abstract |
| Force time measures of beginning and skilled skateboarders performing an 0llie | Walsh, M. and Creekmur, C. and Wojcik, J. | 2006 | Conference Proceeding / Abstract | Quality of evidence lacking. Only a conference proceeding and abstract |
| Visual search behavior in skateboarding | Takeishi, Kento and Tran Thi, Mykha and Tomohisa, Nagano and Takaaki, Kato | 2007 | Peer-reviewed article | No access to the full text article |
| Eye Movements as Indicators of Vestibular Dysfunction | Menshikova, G. Y. and Kovalev, A. I. and Klimova, O. A. and Chernorizov, A. M. | 2015 | Peer-reviewed article | Not relevant for this review. |
| The skateboard speed wobble | Rosatello, M. and Dion, J. L. and Renaud, F. and Garibaldi, L. | 2015 | Peer-reviewed article | Not relevant for this review. Describes the skateboard for use in a mathematical model, but no technical, tactical, or physical components. |
| Stabilising skateboard speed-wobble with reflex delay | Varszegi, B. and Takacs, D. and Stepan, G. and Hogan, S. J. | 2016 | Peer-reviewed article | Not relevant for this review. Describes the skateboard for use in a mathematical model, but no technical, tactical, or physical components. |
| Acceleration helps in skateboarding at high speeds | Varszegi, B. and Takacs, D. and Insperger, T. | 2018 | Peer-reviewed article | Not relevant for this review. Describes the skateboard for use in a mathematical model, but no technical, tactical, or physical components. |
| EEG in motion: Using an oddball task to explore motor interference in active skateboarding | Robles, D. and Kuziek, J. W. P. and Wlasitz, N. A. and Bartlett, N. T. and Hurd, P. L. and Mathewson, K. E. | 2021 | Peer-reviewed article | Not relevant for this review. No technical, tactical, or physical components related to the topic. |
| A cluster analysis and artificial neural network of identifying skateboarding talents based on bio-fitness indicators | Aina Munirah Ab Rasid, Muhammad Zuhaili Suhaimi, Anwar P. P. Abdul Majeed, Mohd Azraai Mohd Razman, Mohd Hasnun Arif Hassan, Nasree Najmi, Noor Azuan Abu Osman & Rabiu Muazu Musa | 2023* | Conference Proceeding / Abstract | Quality of evidence lacking. Only a conference proceeding and abstract and no access to full-text |
| Identification of optimal movement patterns for energy pumping | Micha Luginbühl, Micah Gross, Silvio Lorenzetti, David Graf & Martin J. Bünner | 2023* | Peer-reviewed article | Not relevant for this review. Describes the skateboard and motion for use in a mathematical model, but no technical, tactical, or physical components. |
| * Study was found in the updated search | | | | |

# REFERENCES

1. Walker T. Skateboarding as Transportation: Findings from an Exploratory Study: Portland State University; 2013.

2. Kellett P, Russell R. A comparison between mainstream and action sport industries in Australia: A case study of the skateboarding cluster. Sport Management Review (Elsevier Science). 2009;12(2):66-78.

3. Ellmer EMM, Rynne SB. Professionalisation of action sports in Australia. Sport in Society. 2019;22(10):1742-57.

4. Ellmer E, Rynne S, Enright E. Learning in action sports: A scoping review. European Physical Education Review. 2019;26(1):263-83.

5. Davidson J. Sport and modern technology: the rise of skateboarding, 1963-1978. Journal of Popular Culture. 1985 Spring;18(4):145-57.

6. Huber PA, inventor Skateboard and Accessory. United States. 1979 20 February 1979.

7. Groh BH, Fleckenstein M, Kautz T, Eskofier BM. Classification and visualization of skateboard tricks using wearable sensors. Pervasive and Mobile Computing. 2017;40:42-55.

8. Commission WS. Competition Rules: Olympic Qualification Season 2021. In: Skate W, editor.; 2021. p. 36.

9. Hughes MD, Bartlett RM. The use of performance indicators in performance analysis. Journal of Sports Sciences. 2002 Oct;20(10):739-54.

10. Bäckström Å, Blackman S. Skateboarding: From Urban Spaces to Subcultural Olympians. Young. 2022;30(2):11.

11. Klingner FC, Klingner FP, Elferink-Gemser MT. Riding to the top – A systematic review on multidimensional performance indicators in surfing. International Journal of Sports Science & Coaching. 2021.

12. Arksey H, O'Malley L. Scoping studies: towards a methodological framework. International Journal of Social Research Methodology. 2005;8(1):19-32.

13. Peters MDJ, Godfrey C, McInerney P, Khalil H, Larsen P, Marnie C, et al. Best practice guidance and reporting items for the development of scoping review protocols. JBI Evid Synth. 2022 Feb 9.

14. Tricco AC, Lillie E, Zarin W, O'Brien KK, Colquhoun H, Levac D, et al. PRISMA Extension for Scoping Reviews (PRISMA-ScR): Checklist and Explanation. Annals of Internal Medicine. 2018 2018/10/02;169(7):467-73.

15. The Holy Stoked Collective. The five avatars of skateboarding. [Article] 2020 29 October 2020 [cited 2022 8 March 2022]; Available from: <https://www.redbull.com/us-en/skateboarding-styles-and-disciplines#:~:text=Street%2C%20vert%2C%20downhill%20slide%2C,disciplines%20carved%20out%20of%20skateboard>.

16. Pollock D, Tricco AC, Peters MDJ, McInerney PA, Khalil H, Godfrey CM, et al. Methodological quality, guidance, and tools in scoping reviews: a scoping review protocol. JBI Evid Synth. 2021 Aug 25.

17. Moher D, Shamseer L, Clarke M, Ghersi D, Liberati A, Petticrew M, et al. Preferred reporting items for systematic review and meta-analysis protocols (PRISMA-P) 2015 statement. Systematic Reviews. 2015 2015/01/01;4(1):1.

18. Team TE. EndNote. EndNote X9 ed. Philadelphia, PA: Clarivate; 2013.

19. Mourad Ouzzani HH, Zbys Fedorowicz, and Ahmed Elmagarmid. Rayyan — a web and mobile app for systematic reviews. Systematic Reviews 2016;5(1):210.

20. Cust EE, Sweeting AJ, Ball K, Robertson S. Machine and deep learning for sport-specific movement recognition: a systematic review of model development and performance. Journal of Sports Sciences. 2019 Mar;37(5):568-600.

21. Peters MDJ, Marnie C, Tricco AC, Pollock D, Munn Z, Alexander L, et al. Updated methodological guidance for the conduct of scoping reviews. JBI Evid Implement. 2021 Mar;19(1):3-10.

22. Skate W. Skateboarding Judging Criteria: Olympic Qualification Season 2021. In: Skate W, editor.; 2021. p. 9.

23. Rasid AMA, Kamarudin NA, Abdullah MA, Ibrahim MAR, Shapiee MNAB, Razman MAM, et al. Development of Skill Performance Test for Talent Identification in Amateur Skateboarding Sport. In: Mat Jizat JA, Khairuddin IM, Mohd Razman MA, Ab. Nasir AF, Abdul Karim MS, Jaafar AA, et al., editors. Advances in Robotics, Automation and Data Analytics; 2021 2021//; Cham: Springer International Publishing; 2021. p. 385-90.

24. Ou YK, Chen ZW, Yeh CN. Postural control and functional ankle stability in professional and amateur skateboarders. Healthcare (Switzerland). 2021 2021;9(8).

25. Nakashima M, Chida Y. Simulation study to elucidate the mechanism of ollie jump in skateboarding. Mechanical Engineering Journal. 2021;8(5):21-00230-21-.

26. Furr HN, Nessler JA, Newcomer SC. Characterization of heart rate responses, duration, and distances travelled in youth participating in recreational skateboarding at community skateparks. Journal of Strength & Conditioning Research. 2021 2021;35(2):542-8.

27. Clark LDD, Bishop C, Maloney SJ. Relationships between jumping asymmetries and performance in skateboarders. Journal of Australian Strength & Conditioning. 2021 2021;29(1):13-9.

28. Wood LB, Oliveira A, Santos K, Rodacki A, Lara J. 3D Kinematic Analysis of the Ollie Maneuver on the Skateboard. Apunts: Educacion Fisica y Deportes. 2020 2020(141):87-91.

29. Wiles T, Kellogg D, Furr H, Nessler JA, Newcomer SC. Characterization of adult heart rate responses during recreational skateboarding at community skateparks. International Journal of Exercise Science. 2020 2020;13(2):501-10.

30. Pietta-Dias C, Ruas CV, Bortoluzzi R, Radaelli R, Minozzo F, Pinto RS, et al. Knee side-to-side strength asymmetry and hamstring-to-quadriceps strength ratios in professional street skateboarding athletes. Science & Sports. 2020 2020;35(1):55-7.

31. Leuchanka A, Ewen J, Cooper B. Bipedal in-shoe kinetics of skateboarding–the ollie. Footwear Science. 2017 2017;9:S122-S4.

32. Klostermann A, Küng P. Gaze Strategies in Skateboard Trick Jumps: Spatiotemporal Constraints in Complex Locomotion. Research Quarterly for Exercise & Sport. 2017 2017;88(1):101-7.

33. Nessler JA, Lundquist AL, Jimenez NC, Newcomer SC. Heart Rate Response and Locomotor Activity of Female Skateboarders, BIPOC Skateboarders, and Non-skateboard Users During a Typical Session at a Community Skatepark. International Journal of Exercise Science. 2023;16(7):14.

34. Pham BT. The Biomechanics and Energetics of Skateboarding: University of Colorado; 2016.

35. Vorlíček M, Svoboda Z, Procházková M. Analysis of muscle activity in various performance levels of Ollie jumps in skateboarding: A pilot study. Acta Gymnica. 2015 2015;45(1):41-4.

36. Cesari P, Camponogara I, Papetti S, Rocchesso D, Fontana F. Correction: Might as well jump: Sound affects muscle activation in skateboarding (PLOS ONE (2014) 9, 6 (e100976)). PLoS ONE. 2014 2014;9(6).

37. Candotti CT, Loss JF, Silva RE, Melo MdO, Teixeira RB, Delwing GB, et al. Lower limb force, power and performance in skateboarding: an exploratory study. Revista Brasileira de Ciências do Esporte. 2012;34:697-711.

38. Hetzler RK, Hunt I, Stickley CD, Kimura IF. Selected Metabolic Responses to Skateboarding. Research Quarterly for Exercise & Sport. 2011;82(4):788-93.

39. Determan JJ, Frederick EC, Cox JS, Nevitt MN. High impact forces in skateboarding landings affected by landing outcome. Footwear Science. 2010 2010;2(3):159-70.

40. Nevitt M, Determan J, Felix A, Cox J. Frictional requirements of skateboarding shoes during a push-off. Footwear Science. 2009;1:34-5.

41. Frederick EC, Determan JJ, Whittlesey SN, Hamill J. Biomechanics of Skateboarding: Kinetics of the Ollie. Journal of Applied Biomechanics. 2006 2006;22(1):33-40.

42. Helsing D. Skateboarding and the City: A Complete History. Journal of Popular Culture. 2020;53(5):1216-7.

43. McKay AKA, Stellingwerff T, Smith ES, Martin DT, Mujika I, Goosey-Tolfrey VL, et al. Defining Training and Performance Caliber: A Participant Classification Framework. Int J Sports Physiol Perform. 2022 Feb 1;17(2):317-31.

44. Rodríguez-Rivadulla A, Saavedra-García MÁ, Arriaza-Loureda R. Skateboarding Injuries in Spain: A Web-Based Survey Approach. Orthopaedic journal of sports medicine. 2020;8(3):2325967119884907.

45. McAlpine PR. Biomechanical Analysis of Snowboard

Jump Landings:

A Focus on the Ankle Joint Complex: The University of Auckland; 2010.

46. Künzell S, Lukas S. Facilitation effects of a preparatory skateboard training on the learning of snowboarding. Kinesiology. 2011;43(1.):56-63.

47. Hoholm SL. “Pop” and its relation to performance factors and equivalent fall height in World Cup slopestyle for skiers and snowboarders: Norwegian School of Sport Sciences; 2022.

48. Farley ORL, Harris NK, Kilding AE. Physiological demands of competitive surfing. Journal of Strength & Conditioning Research. 2012;26(7):1887-96.

49. Vernillo G, Pisoni C, Thiébat G. Physiological and Physical Profile of Snowboarding: A Preliminary Review. Front Physiol. 2018;9:770.

50. Haugen TA, Breitschädel F, Wiig H, Seiler S. Countermovement Jump Height in National-Team Athletes

of Various Sports: A Framework for Practitioners and Scientists. International Journal of Sport Physiology and Performance. 2021;16:6.

51. Tran TT, Lundgren L, Secomb J, Farley ORL, Haff GG, Seitz LB, et al. Comparison of Physical Capacities Between Nonselected and Selected Elite Male Competitive Surfers for the National Junior Team. International Journal of Sports Physiology & Performance. 2015;10(2):178-82.

52. Dowse RA, Secomb JL, Bruton M, Nimphius S. Ankle proprioception, range of motion and drop landing ability differentiates competitive and non-competitive surfers. Journal of Science & Medicine in Sport. 2021;24(6):609-13.

53. Löfquist I, Björklund G. What magnitude of force is a slopestyle skier exposed to when landing a big air jump? Int J Exerc Sci. 2020;13(1):11.

54. Marinšek M. Basic landing characteristics and their application in artistic gymnastics. Science of Gymnastics Journal. 2016;2(2):9.

55. Wheaton B, Thorpe H. Action Sports, the Olympic Games, and the Opportunities and Challenges for Gender Equity: The Cases of Surfing and Skateboarding. Journal of Sport & Social Issues. 2018;42(5):315-42.

1. If not explicitly stated, equipment use was up to the participant and not controlled for or measured [↑](#footnote-ref-1)
2. The lead author ranked each participant by their ‘skateboarding ability’, however no details surrounding the actual judged skateboarding were provided (when they skated, what tricks or how many they performed, style of skateboarding, etc.) [↑](#footnote-ref-2)
